# The Performance of Vascular Age in the Assessment of Cardiovascular Risk of Patients with Rheumatoid Arthritis

**DOI:** 10.3390/jcm9124065

**Published:** 2020-12-16

**Authors:** Iván Ferraz-Amaro, Alfonso Corrales, Juan Carlos Quevedo-Abeledo, Belén Atienza-Mateo, Diana Prieto-Peña, Ricardo Blanco, Javier Llorca, Miguel Á. González-Gay

**Affiliations:** 1 Division of Rheumatology, Hospital Universitario de Canarias, 38320 Tenerife, Spain; 2 Division of Rheumatology, Hospital Universitario Marqués de Valdecilla, Universidad de Cantabria, 39008 Santander, Spain; afcorralesm@hotmail.com (A.C.); mateoatienzabelen@gmail.com (B.A.-M.); diana.prieto.pena@gmail.com (D.P.-P.); ricardo.blanco@scsalud.es (R.B.); miguelaggay@hotmail.com (M.Á.G.-G.); 3 Epidemiology, Genetics and Atherosclerosis Research Group on Systemic Inflammatory Diseases, IDIVAL, 39011 Santander, Spain; 4 Division of Rheumatology, Hospital Doctor Negrín, 35010 Las Palmas de Gran Canaria, Spain; quevedojcarlos@yahoo.es; 5 University of Cantabria, 39011 Santander, Spain; llorcaj@unican.es; 6 CIBER Epidemiología y Salud Pública (CIBERESP), 39011 Santander, Spain; 7 Cardiovascular Pathophysiology and Genomics Research Unit, Faculty of Health Sciences, School of Physiology, University of the Witwatersrand, Johannesburg 2193, South Africa

**Keywords:** vascular age, rheumatoid arthritis, cardiovascular risk assessment, carotid plaque

## Abstract

**Background.** Cardiovascular (CV) disease risk prediction models developed for use in the general population have suboptimal performance in patients with rheumatoid arthritis (RA). Vascular age (VA) is a new concept that has been proposed as a measure of CV ‘relative’ risk instead of the ‘absolute’ risk that current prediction models provide. In the present study we aim to study the performance of vascular age (VA) in the assessment of CV risk in patients with RA. We additionally aimed to analyze its relation with subclinical atherosclerosis as measured through carotid plaque ultrasound. **Methods.** A total of 1173 non-diabetic RA patients without previous CV events were included. Disease characteristics, SCORE, VA determined on SCORE and on carotid intima media thickness (cIMT), and the presence of plaque through carotid ultrasound were assessed. The interrelations of VA with SCORE, and its associations with subclinical carotid atherosclerosis were studied. **Results.** On average, RA patients had both a SCORE determined VA (4.7 years) and a cIMT-based VA (2.4 years) significantly higher than the chronological age. When these differences were analyzed in different age intervals, while VA based on SCORE was significantly higher compared to chronological age in all age ranges, VA determined on cIMT was significantly elevated only in RA patients younger than 60 years. The area under the curve analysis for the association of SCORE and VA with the presence of carotid plaque disclosed no differences between both parameters. VA was associated with the presence of carotid plaque after multivariable regression analysis in patients younger than 60 years old. **Conclusion.** VA is significantly higher than chronological age in patients with RA. The performance of VA in its relation to carotid plaque is similar to that of the SCORE.

## 1. Introduction

Rheumatoid arthritis (RA) is a chronic inflammatory arthritis associated with systemic complications. In this sense, the mortality of patients with RA is higher than that of the general population. This increased risk of premature death in patients with RA is largely due to cardiovascular disease (CVD), particularly coronary artery disease. In this sense, it has been estimated that the risk of coronary artery disease mortality is 59% higher in patients with RA than in the general population [1]. Interestingly, it has been shown that CV disease risk prediction models like Framingham Risk Score or the Systematic Coronary Risk Evaluation (SCORE) algorithm, that were originally developed for use in the general population and rely heavily on traditional CVD risk factors, have suboptimal performance in patients with RA and underestimate CV risk [2,3]. For example, a recent study undertaken to assess the predictive ability of four established CV risk models for the 10-year risk of fatal and non-fatal CV diseases in patients with RA established that these risk models generally underestimate (SCORE, Framingham Risk Score, Reynolds risk score) or overestimate (QRisk II) CV risk in patients with RA [2].

In 2010, a new cardiovascular risk table from the SCORE project was published, which incorporated the new concept ‘vascular age’ [4]. Since then, vascular age (VA) (also known as heart age or CV risk age) has been defined as another indicator suitable for patients in general population. The VA of a patient with CV risk factors is defined as the age that an individual of the same sex as a given patient should be if he/she had the same absolute risk but controlled risk factors. VA is independent of the CV endpoint used. This fact makes it possible to avoid the dilemma of using a risk estimation system based on CV mortality or total CV events. Furthermore, VA can be used in any population independently of the baseline risk and secular changes in mortality. Consequently, it avoids the necessity of recalibration [5]. Similarly, another VA based on carotid intima media thickness (cIMT) has been proposed. cIMT determined VA (iVA) is defined as the age at which the composite value of cIMT for an individual of a given gender would represent the median value (50th percentile) of cIMT. This is of great help in establishing the age component of the population-based CV disease risk estimate [6].

The performance of VA (based both on SCORE and on cIMT) in the assessment of CV risk in patients with RA has not been explored before. Moreover, the relation of VA with subclinical atherosclerosis in patients with RA is pending to be investigated.

## 2. Materials and Methods

### 2.1. Patients Included in the Study

A total of 1173 patients with RA were evaluated in a cross-sectional study. Patients had to be 18 years old or older. Additionally, for inclusion, they had to meet the 2010 ACR/EULAR classification criteria for RA [7]. Moreover, disease duration had to be ≥1 year. Those taking prednisone, or an equivalent dose at a dose >10 mg/day were excluded. Patients with diabetes, history of malignancy, active infection or any other chronic disease, including those with a glomerular filtration rate <60 mL/min/1.73 m^2^ were also excluded. The Institutional Review Committees at Hospital Universitario de Canarias, Hospital Doctor Negrín, and Hospital Marqués de Valdecilla, Spain, study protocol approved the study (Approval reference: 17/2012). Additionally, informed consent was requested from all the patients

### 2.2. Laboratory Assessment and Data Collection

In addition to the physical examination, a comprehensive evaluation of cardiovascular risk factors and medication use was performed. Weight, height, body mass index, waist-to-hip ratio, and systolic and diastolic blood pressure were evaluated. Additionally, history of smoking (current smoker vs. non-smoker), diabetes, and hypertension was obtained. Information on diagnoses and medications were double-checked. We defined the presence of dyslipidemia if the patient had one of the following data: total cholesterol >200 mg/dL, triglyceride >150 mg/dL, HDL cholesterol <40 in men or <50 mg/dL in women, or LDL cholesterol >130 mg/dL. Cholesterol, triglycerides, and HDL cholesterol were determined by the enzymatic colorimetric assay. LDL cholesterol was calculated using the Friedewald formula. The erythrocyte sedimentation rate (ESR) and high-sensitivity C-reactive protein (CRP) were determined using standard techniques. To establish activity of the disease, we used the using the Disease Activity Score (DAS28) in 28 joints [8], the Clinical Disease Activity Index (CDAI) [9] and the Simple Disease Activity Index (SDAI) [10]. As previously reported, clinical remission was defined as DAS28 being lower than 2.6. Low disease activity if DAS28 ranged between 2.6 and 3.2. Moderate disease activity if DAS28 was >3.2 but not higher than 5.1. Very high disease activity was considered to be present if DAS28 was >5.1 [11].

As previously described, we calculated the Systemic Coronary Risk Estimation (SCORE) [12]. Additionally, as previously defined, VA equation was determined by equating the generic formula for 10-year CVD risk (with unknown risk factor levels) to the generic formula for 10-year CVD risk in a person with age = X and ideal risk factor levels (total cholesterol 4 mmol/L, systolic blood pressure 120 mm Hg, and non-smoker) and solving for X [5]. The vascular age determined by cIMT (iVA) was established by linear regression modeling using the previously reported cIMT percentiles (5th, 10th, 25th, 50th, 75th, 90th, and 95th) of the Spanish population according to chronological age and gender [13]. Linear regression model was built for each of the cIMT percentile functions for the common carotid arterial segment, by gender (male and female) and age (5-year increments from 35–80 years old). Composite cIMT values were used to establish the iVA, defined as the age at which the composite cIMT value for an individual of a given gender would represent the median value (50th percentile) for the Spanish population.

### 2.3. Carotid Ultrasound Evaluation

In all patients, a carotid ultrasound assessment was performed to determine the carotid intima-media thickness (cIMT) in the common carotid artery and to identify the presence of plaques in the extracranial carotid tree. As previously described, a commercially available scanner, the Esaote Mylab 70 (Genoa, Italy), equipped with a 7–12 MHz linear transducer and an automated software-guided radiofrequency technique, Quality Intima Media Thickness in real-time (QIMT, Esaote, Maastricht, Holland), was used. As described in previous reports of our group [14,15], plaque criteria in the accessible extracranial carotid tree (common carotid artery, bulb and internal carotid artery) were as follows: a focal protrusion in the lumen measuring at least cIMT >1.5 mm; a protrusion at least 50% greater than the surrounding cIMT; or arterial lumen encroaching >0.5 mm [16].

### 2.4. Statistical Analysis

Binary variables shown as frequencies were used to describe demographic and clinical characteristics. Continuous variables data are shown as mean ± standard deviation (SD) or as a median and interquartile range (IQR) for non-normally distributed variables. Student’s t–test for one sample was used to compared age differences (VA minus chronological age) with null hypothesis (differences equal to zero). Classification performance of the SCORE and VA with respect to the presence of carotid plaques was analyzed by area under the curve (AUC) analysis (Receiver Operating Characteristics). Differences between AUCs were calculated using the DeLong method [17]. Multivariable logistic regression, adjusted for age at debut and disease duration, was used to study the relation between VA and the presence of carotid plaque. All analyses used a 5% two-sided significance level and were performed using SPSS software, version 21 (IBM, Chicago, IL, USA) and STATA software, version 13/SE (Stata Corp., College Station, TX, USA). A *p*-value < 0.05 was considered statistically significant.

## 3. Results

### 3.1. Demographic, Laboratory and Disease-Related Data

A total of 1173 patients with RA were included in this study. The demographic and disease-related characteristics of the participants are shown in Table 1. Mean ± SD age was 57 ± 12 years and 78% of the patients were female. Each CV risk factor (current smoking, hypertension, dyslipidemia and obesity) was present in at least one quarter of the patients. None of the patients had diabetes. ESR and CRP were, respectively, 13 (IQR 6–25) mm/1st h and 2.6 (IQR 0.9–6.3) mg/L.

Average disease duration was 4 (IQR 1–10) years and age of onset of the disease was 49 ± 14 years old. RA patients had moderate disease activity as shown by the mean DAS28 (3.05 ± 1.47). Almost half of the patients (48%) were taking prednisone (the median dose of those patients on prednisone was 5 (IQR 3–6) mg/day at the time of the study). Fifty-nine percent of patients were found to be positive for rheumatoid factor and 54% for anti-citrullinated protein antibody. Besides, 77% were on disease-modifying antirheumatic drugs and 13% were receiving anti-TNF-alpha therapy. Additional information regarding RA patient characteristics is shown in Table 1.

### 3.2. Vascular Age and cIMT-Based Vascular Age Relation to Chronological Age

**Figure 1:**
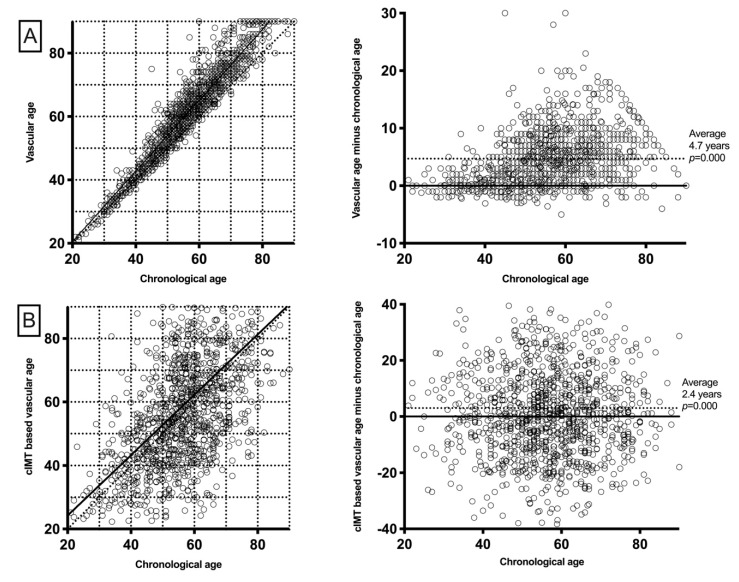
Vascular age relation with chronological age. (**A**) As both VA and chronological ages rise, patients with higher chronological age exhibit a higher difference between the ages. Moreover, average VA is significantly higher than chronological age. (**B**) The difference between iVA and chronological age is higher in younger patients and this difference decreases as age increases. Average iVA is significantly higher than chronological age.

The mean VA was 61.5 ± 14.7 years (IQR 49–65), which represented an average increase of 4.7 ± 5.0 years (IQR 1.0–7.1 years) over chronological age (*p* < 0.001). Similarly, iVA was 59.2 ± 21.3 years (IQR 44.1–71.2 years). This represented, on average, 2.4 ± 17.7 years (IQR −9.2–11.8) higher compared to chronological age (Figure 1). When these differences were assessed in different age intervals, similar results were found for VA. In this sense, in all age strata (<40, 40–50, 50–60, 60–70, and >80), VA was significantly higher compared to chronological age. These differences were found to increase as chronological age increased. To the contrary, iVA was found to be significant higher only in younger age intervals but not in patients older than 60 years (Table 2).

Vascular age, based on both SCORE and cIMT, was not associated with disease duration or disease activity score.

### 3.3. VA Performance for the Presence of Carotid Plaque

SCORE was associated with the presence of carotid plaque in all age intervals (Table 3). In this sense, AUCs were statistically significant throughout these age ranges. Similarly, VA, which has a nonlinear relation with SCORE, disclosed significant values in its AUC analysis for the presence of carotid plaque. Moreover, when AUC were compared between SCORE and VA, neither was different, therefore showing comparable discrimination for the presence of carotid plaque.

The relation of VA with carotid plaque, adjusted for age at disease onset and disease duration, was additionally assessed. In this sense, VA was associated with carotid plaque only in patients in the range between 40 and 60 years old (Table 3).

## 4. Discussion

Vascular age has recently emerged for CV disease risk assessment and can either be computed using conventional risk factors or by using cIMT derived from carotid ultrasound. According to our results, VA is higher compared to chronological age in patients with RA. Moreover, VA performance in the discrimination of carotid plaque is equivalent to that achieved by SCORE. Our findings demonstrate that VA assessment, a more intuitive approach for the understanding of CV risk in general population, is applicable in patients with RA and is similar to the one that SCORE discloses. Moreover, VA is related to subclinical atherosclerosis in young RA patients.

VA has only been assessed in RA patients once in the literature. In this study of 1974 patients followed-up over 5 years, a VA model was found to have comparable performance in predicting CV events in RA patients compared to SCORE [18]. Our study, that used carotid plaque as outcome, confirmed these results.

Absolute risk estimation by SCORE is known to be driven primarily by age. This mean that a young person with a very high relative risk because of multiple CV risk factors may still be in a low SCORE absolute risk. The fact that VA was associated in our study with carotid plaque in patients younger than 60 years old is of great importance. Consequently, the estimation of VA would be highly recommended in young patients with RA in whom the calculation of the absolute risk SCORE would underestimate the actual CV risk but in whom there is an association with subclinical atherosclerosis.

Furthermore, one of the concerns about the use of conventional CV risk algorithms is that they assign the same number of points to each patient at a given age regardless of their atherosclerotic burden, which ignores the large variation in plaque burden or cIMT. at any chronological age. Of note, VA cannot be assessed using carotid plaque because this is a binary variable in which linear equations cannot be derived. However, VA based on cIMT is feasible. This implies that cIMT provides an opportunity to adjust a patient’s chronological age for their atherosclerotic burden. There are several studies that have demonstrated that cIMT predicts future cardiovascular events in the general population [19] and also in patients with RA [20]. Moreover, cIMT has been found to be useful in improving risk discrimination in the Framingham Risk Score, and that substituting chronological age for cIMT-determined vascular age may improve individual cardiovascular risk prediction [6,21]. Our work is the first in the literature in which iVA was assessed in patients with RA. According to our results, iVA was higher compared to chronological age in patients with RA. This means that cIMT measurement by carotid ultrasound provides a clinical tool that can allow us to individualize risk assessment and optimize patient care in patients with RA.

CV risk is a mathematical concept that some people do not understand well. It is difficult to make a young subject understand why their risk SCORE of a certain percentage is alarming. We think that patients will understand their current situation if their doctors explain that their heart and arteries are a few years older than their actual age. This information could help patients to improve their adherence to drug treatment and lifestyle modifications recommended by their physicians [22]. Recently, a randomized clinical trial of 3000 people compared three different strategies: standard clinical care without information on vascular or VA risk (control group), clinical care with information on the individual’s absolute risk, and clinical care with information on the individual’s VA [23]. The change in risk factors was assessed after 12 months. The group that showed the greatest improvement (statistically significant) in all risk factors was the group informed about VA, showing the greatest decrease in their CV risk. We believe that the fact that, in our study, VA was associated with carotid plaque in younger patients should compel us to treat our RA patients by informing them of their VA.

It should be noted that VA is derived from the SCORE formula. This would imply that the relationship between SCORE and VA would be expected to exist. However, this is not the case, because the relationship between them is not linear, but exponential. For this reason, it should not be accepted that the relationship between SCORE and carotid plaque is the same as that between SCORE and VA. On the contrary, the relationship between SCORE and iVA is linear as previously explained. For this reason, the relationship between iVA and SCORE has not been assessed in our work.

In conclusion, VA is an adequate tool for CV risk assessment in patients with RA. Due to the fact that VA is generally higher than chronological age, this can be an intuitive and effective way to convey our concern about CV risk in patients with RA. Furthermore, cIMT assessment can be used in RA patients to adjust the SCORE risk estimate. More research is still needed to estimate the true CV risk in the RA population.

## Figures and Tables

**Table 1:** Characteristics of rheumatoid arthritis patients.

	RA Patients
	(*n* = 1173)
Age, years	57 ± 12
Female, *n* (%)	917 (78)
Body mass index, kg/m^2^	28 ± 5
Cardiovascular co-morbidity	
Smoking, *n* (%)	307 (26)
Diabetes, *n* (%)	0 (0)
Hypertension, *n* (%)	285 (24)
Dyslipidemia, *n* (%)	290 (25)
Obesity, *n* (%)	327 (28)
Statins, *n* (%)	252 (21)
Laboratory and lipid profile	
ESR, mm/1st h	13 (6–25)
CRP, mg/L	2.6 (0.9–6.3)
Cholesterol, mg/dL	205 ± 36
Triglycerides, mg/dL	112 ± 63
HDL cholesterol, mg/dL	61 ± 17
LDL cholesterol, mg/dL	121 ± 31
Atherogenic index	3.57 ± 1.06
Rheumatoid arthritis related data	
Disease duration, years	4 (1–10)
Age of onset, years	49 ± 14
DAS28	3.05 ± 1.47
DAS28-PCR	2.81 ± 1.37
SDAI	10 (5–18)
CDAI	9 (4–16)
Rheumatoid factor, *n* (%)	694 (59)
ACPA, *n* (%)	634 (54)
Prednisone intake, *n* (%)	562 (48)
Current prednisone dose *, mg/day	5 (3–6)
NSAIDs intake, *n* (%)	471 (40)
DMARDs, *n* (%)	909 (77)
Methotrexate, *n* (%)	683 (58)
Leflunomide, *n* (%)	131 (11)
Hydroxychloroquine, *n* (%)	254 (22)
Anti TNF therapy, *n* (%)	158 (13)
Abatacept, *n* (%)	14 (1)
Tocilizumab, *n* (%)	59 (5)
Rituximab, *n* (%)	21 (2)
Tofacitinib, *n* (%)	11 (1)
Baricitinib, *n* (%)	9 (1)
Carotid assessment	
cIMT, microns	690 ± 140
Carotid plaque, *n* (%)	587 (50)

Data represent means ± SD or median (IQR) when data were not normally distributed; CRP: C reactive protein; LDL: low-density lipoprotein; DMARD: disease-modifying antirheumatic drug; ESR: erythrocyte sedimentation rate; DAS: disease activity score; HDL: high-density lipoprotein; SDAI: Simplified Disease Activity Index; CDAI: Clinical Disease Activity Index; TNF: anti-nuclear tumor factor; ACPA: Anti-citrullinated protein antibody; cIMT, carotid intima media thickness. NSAIDs: Nonsteroidal anti-inflammatory drugs. * In those patients taking prednisone.

**Table 2:** Vascular ages and chronological age intervals.

cIMT Based Vascular Age					
Age	*n*	cIMT, Microns	cIMT Vascular Age, Years	Age Difference, Years	*p*
<40	115	538	36 ± 13	1 (−5–10)	0.055
40–50	223	619	49 ± 15	1 (−6–10)	**0.005**
50–60	393	690	60 ± 19	2 (−8–14)	**0.000**
60–70	273	725	65 ± 19	−1 (−14–11)	0.916
>80	168	811	78 ± 20	−1 (−12–15)	0.270
SCORE-based vascular age					
Age	*n*	SCORE, %	Vascular age, years	Age difference, years	*p*
<40	115	0.0 (0.0–0.0)	35 ± 7	0 (−1–2)	**0.030**
40–50	223	0.2 (0.1–0.4)	49 ± 5	2 (0–5)	**0.000**
50–60	393	1.0 (0.6–1.7)	61 ± 6	5 (2–8)	**0.000**
60–70	273	3.0 (1.8–4.2)	71 ± 6	5 (3–9)	**0.000**
>80	168	8.0 (5.1–12.8)	83 ± 6	6 (3–9)	**0.000**

Age difference refers to the difference between vascular ages (SCORE- or cIMT-based) and chronological age. *p* refers to the significance of the comparison of ‘age difference’ and 0. SCORE: Systematic Coronary Risk Evaluation; cIMT: carotid intima media thickness. Significant *p* values are depicted in bold.

**Table 3:** SCORE and VA performance for the presence of carotid plaque.

Age	*n*	SCORE, %	Carotid Plaque, *n*	SCORE AUC for Carotid Plaque	Vascular Age AUC for Carotid Plaque	*p*	OR (95% CI), *p*
<40	115	0.0 (0.0–0.0)	4	-	-	-	
40–50	223	0.2 (0.1–0.4)	45	0.711 (0.624–0.797)	0.690 (0.605–0.776)	0.40	**1.19 (1.09–1.30), 0.000**
50–60	393	1.0 (0.6–1.7)	201	0.597 (0.541–0.653)	0.596 (0.541–0,652)	0.97	**1.07 (1.02–1.12), 0.006**
60–70	273	3.0 (1.8–4.2)	188	0.590 (0.520–0.660)	0.581 (0.510–0.651)	0.49	1.04 (0.98–1.10), 0.16
>80	168	8.0 (5.1–12.8)	149	0.690 (0.588–0.794)	0.710 (0.607–0.813)	0.24	1.07 (0.96–1.20), 0.24

AUC: area under the curve; SCORE: Systematic Coronary Risk Evaluation. No analyses were performed for <40 patients because only four patients in this group had carotid plaque. *p* refers to the comparison of AUC between SCORE and vascular age. VA odds ratios for carotid plaque are adjusted for disease duration and age at debut. Significant *p* values are depicted in bold.
